# Comparative Study of Friction and Wear Performance of PEK, PEEK and PEKK Binders in Tribological Coatings

**DOI:** 10.3390/polym14194008

**Published:** 2022-09-25

**Authors:** Judith M. Pedroso, Marco Enger, Pedro Bandeira, Fernão D. Magalhães

**Affiliations:** 1 GGB Bearings Technology Porto, Rua Dr. Júlio de Matos, 828/882, 4200-355 Porto, Portugal; 2 LEPABE— Faculty of Engineering, University of Porto, Rua Dr. Roberto Frias, 4200-465 Porto, Portugal; 3 AliCE—Associate Laboratory in Chemical Engineering, Faculty of Engineering, University of Porto, Rua Dr. Roberto Frias, 4200-465 Porto, Portugal; 4 GGB Bearings Technology GmbH, 74078 Heilbronn, Germany

**Keywords:** coating, PEEK, PEK, PEKK, PTFE, tribology, friction, wear

## Abstract

Tribological coatings are widely used in industry, particularly when the conventional oil lubrication of sliding surfaces has to be replaced by maintenance-free contacts. This work studies the tribological performance of waterborne tribological coatings based on three binders of the polyaryletherketone (PAEK) family: polyetherketone (PEK), PEEK, and polyetherketoneketone (PEKK). Even though PEEK is a well-known commercial solution for this type of tribological coatings, PEK and PEKK have never been studied in such a context. PTFE particles were added to all coatings as a solid lubricant. High thermal resistance of the binder materials was confirmed, with decomposition starting above 550 °C, under either N_2_ or O_2_ atmosphere. XRD analysis showed that PEK and PEEK are semi-crystalline after being subjected to the coating curing conditions, while PEKK is amorphous. The coatings were successfully applied with thicknesses of 20–30 µm. Tribological measurements showed that the PEK-based coating possesses a coefficient of friction (COF) of 0.08 under high load and pressure conditions (hertzian point contact), which is lower than the reference PEEK-based coating (around 0.11). The PEKK-based coating showed an impressive wear resistance with almost no wear measured compared to the 105 µm wear obtained for PEEK-based coating, while showing a similar COF. These results suggest that PEK and PEKK seem to be interesting alternatives to PEEK and should be further studied for use in tribological coatings.

## 1. Introduction

Polyaryletherketone (PAEK) is a family of semi-crystalline thermoplastic polymers, synthesized via Friedel–Crafts acylation, able to cope with high temperatures and high mechanical stress conditions. Their chemical structure contains phenylene rings bonded by oxygen functional groups (ether and carbonyl ketonic groups) [1,2]. The presence of aryl rings in the molecular chain contributes to the rigidity of the material, whereas the ether groups provide flexibility to the overall polymeric chain [3]. 

**Figure 1:**
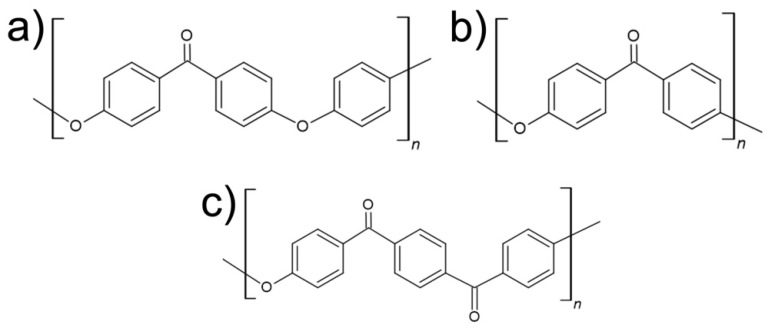
Chemical structures of (**a**) PEEK—Polyetheretherketone, (**b**) PEK—Polyetherketone and (**c**) PEKK—Polyetherketoneketone.

Polyetheretherketone (PEEK) is one of the better-known polymers in the PAEK family. The repeating unit is constituted by two ether groups and one carbonyl group (ketone), as shown in Figure 1a. The combination of high thermal transition temperatures (glass transition temperature around 150 °C and melting temperature around 340 °C) and good mechanical properties make it an interesting choice for uses that request scratch and wear resistance under demanding conditions. However, this polymer has limited applicability in tribological applications when used alone, since its coefficient of friction, COF, is relatively high, reaching about 0.7 under some conditions [4]. High COFs cause the near-contact temperature (on the tribological pair) to increase, reducing the local hardness of the polymer [5,6]. This induces deformation, increasing contact area, and therefore the friction force, resulting in inherent performance instability [7]. Recent works have been directed towards tribological use of PEEK combined with self-lubricating materials, therefore allowing obtaining a usable tribological performance. One such example is the use of PTFE particles, which allows the formation of a transfer layer in the tribo-pair contact, reducing shear stress and therefore controlling frictional heating [8]. This is a standard procedure in dry lubrication conditions (absence of liquid lubricant), such as the ones used in this study. These particles help to form a sliding film on the polymer surface and also support to establishment of a thin and robust transfer film on the counter surface. Both films are crucial for friction and wear stabilization. The final tribological performance therefore results from the combined contribution of the binder and the lubricant filler (PTFE). Most works deal with PEEK/PTFE systems in bulk form [4,9,10,11,12,13]. The effect of other fillers on PEEK’s tribological performance has also been studied, again for the composite in bulk form [14,15,16,17].

Existing literature on PEEK-based thin tribological coatings is more limited. Reported application methods are thermal spraying [18,19,20], electrophoretic deposition [6,21], and electrostatic powder spraying [22,23,24]. Zhu et al. [23] studied the effect of PTFE at different loadings on the mechanical properties of PEEK coatings. In such a system, PEEK acts as the binder, providing a continuous matrix for dispersion of the PTFE particles and ensuring good adhesion to the substrate. The authors concluded that the addition of PTFE did not affect the final hardness of the coating but improved the tribological performance in terms of lowering friction and increasing wear resistance. Tharajak et al. [25] studied the addition of hexagonal boron nitride (hBN) powders to PEEK coatings, observing remarkable reductions in COF and wear rates. 

Tribological coatings based on PEEK are used in industrial applications involving maintenance free sliding surfaces (i.e., absence of liquid lubricants). For example, in the automotive industry, PEEK coatings are applied onto piston skirts to promote a reduction of fuel consumption [26], while PEEK and PEEK/SiC are used as coatings applied to aluminum substrates with a significant reduction in friction and wear of the metal substrate, especially important in general industry mechanisms where an ecological and economical approach is required. [27,28]. Another use is in air-conditioning oil-less compressor surfaces [29]. However, due to the rising prices of this raw material, the industry’s interest on PEEK has been relatively limited. Customers tend to select less expensive alternatives, even though this may result in increased maintenance costs and decreased efficiency. Polymer suppliers have been suggesting that the use of other PAEK-family polymers, besides PEEK, or combinations within this family, can reduce the price by around 30% while maintaining performance in terms of strength and stiffness [30]. This, however, remains to be shown.

Polyetherketone (PEK) is another polymer in the PAEK family. The repeating unit contains one ether functional group and one carbonyl group, as indicated in Figure 1b. This polymer is very similar to PEEK regarding processability by extrusion or injection moulding, and in terms of mechanical and thermal properties [31]. 

Contrary to PEEK, there are very few published studies on the tribological performance of PEK. Gan et al. [32] studied the addition of mica particles (20 wt% loading), observing improvements under high loads. Bijwe et al. [33] studied the tribo-performance of PEK and PEEK, alone and combined with different solid lubricants and fibers. The materials were in bulk form. Both polymers showed similar behavior in terms of friction coefficient, displaying high values (around 0.45). In terms of wear, PEEK showed a better wear rate than PEK until 125 N load. At 150 N load, PEK showed better wear performance. A more recent study, from the same group, was focused on the tribological performance of PEK composites filled with polybenzimidazole (AB-PBI) and compared with commercially available PBI/PEEK composite. The results showed good tribo-performance, with reductions in friction coefficient and wear rate in relation to the PEEK-based system [34]. 

Polyetherketoneketone (PEKK) is also a polymer of the PAEK family. Its repeating unit contains one ether functional group followed by two carbonyl groups, as shown in Figure 1c. PEKK consumption has been rising lately due to its combination of wear and creep resistances, flame retardancy, and compressive strength [3]. It has been recently studied as a biomaterial in dental and medical applications [35]. Published works on its tribological performance are few. Yahiaoui et al. [36] studied the tribological performance of PEKK/steel contact and its impact on acoustic emissions. The authors used a commercially available PEKK grade and prepared the specimens by injection moulding. Gan et al. [37] prepared a composite based on PEKK and mica, showing that the tribological performance is affected by the ratio of filler, being optimal (lower friction coefficient) for about 20 wt.% of mica.

No studies have been reported on the use of PEK or PEKK in tribological coatings. The present work describes the usage of PEK, PEEK, and PEKK as coating binders, combined with PTFE as self-lubricating solid filler, and compares their tribological performance. All coating formulations were waterborne, for environmental and occupational health reasons. 

## 2. Materials and Methods

### 2.1. Materials

Polyetherketone (PEK) and polyetherketone-ketone (PEKK) were supplied by Gharda Chemicals Ltd. (Mumbai, India). Polyetheretherketone (PEEK) was supplied by Solvay Specialty Polymers S.p.A. (Lombardia, Italy). All binders were supplied in powder form. Polytetrafluoroethylene (PTFE), Dyneon TF9201Z micropowder, to be used as a self-lubricating filler was purchased from 3M (Saint Paul, MN, USA). Surfactant/dispersant dioctyl sulfosuccinate sodium salt (DOSS) was purchased from Enzymatic S.A. (Lisbon, Portugal). Deionized water was used for preparing the formulations. All materials were used as received. Table 1 shows some mechanical properties of PEK, PEEK, and PEKK provided by the manufacturers.

### 2.2. Coatings Preparation

For the physical-chemical characterization tasks, coatings were formulated with and without addition of PTFE particles.

For the coating formulations without PTFE: 92.5 g of binder in powder form was firstly dispersed in a 500 mL glass flask, containing 147 g of a mixture of deionized water and DOSS (4 wt.%), for 5 min. A standard blade propeller was used to promote initial deagglomeration. Afterwards, the binder dispersion was homogenized with an IKA (Staufen, Germany) Ultra-Turrax T-18 rotor-stator at 16,000 rpm for 20 min. A cold-water bath was used to maintain the liquid temperature below 30 °C. 

For the coating formulations containing PTFE, 78 g of binder in powder form and 14.5 g of PTFE micropowders were previously mixed in a 250 mL glass flask using a spatula. Then, the powder mixture was dispersed in a 500 mL glass flask, containing 147 g of a mixture of deionized water and DOSS (4wt.%), and the procedure of mixing was the same as previously described for the coating formulations without PTFE.

The final formulations contain around 38 wt.% of solids. The mass fraction of PTFE (based on solid content) is 0.158, which corresponds to a volume ratio of PTFE to binder of 10:90. This ratio was already reported for PEEK/PTFE polymer composites as optimal to obtain a balanced reduction in friction and wear [38].

The substrate of the tribological specimens was 40CrMnMoS8.6 steel, with 80 mm diameter and 10 mm thickness. The coated area had 30 mm diameter, centered in one of the faces. DC04 steel plates with 0.9 mm thickness were also used as substrate for some characterization tests, e.g., tunning spray application parameters and X-ray diffraction analysis.

All the steel parts were previously pyrolyzed at 430 °C for 1 h to remove contaminants. The area to be coated was blasted at high-pressure with angular corundum by Flupol, Surface Engineering S.A (Valongo, Portugal). The blasting parameters were controlled in order to achieve a surface roughness of 2.5 to 3.0 µm Ra.

The coating formulations were applied by a conventional manual spray gun at 1.2 bar pressure for atomization, and allowed to dry for 5 min at 100 °C in a convection oven. 

**Figure 2:**
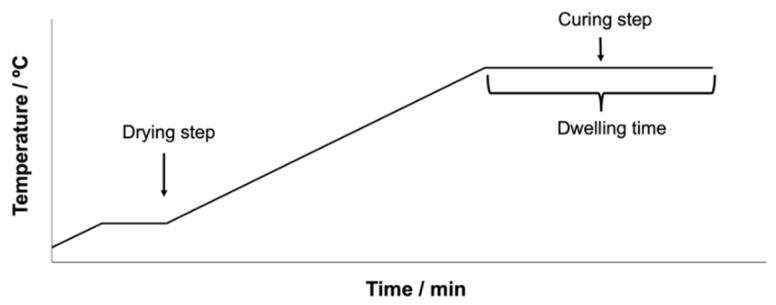
Schematic of the curing cycle performed in the coatings using a convection oven.

The final step was curing, which promotes coalescence of the binder particles and yields a continuous coating. This was performed in a convection oven using a programmed ramp (see Figure 2). The curing cycles were determined by testing the capability of the material to flow and ensuring a stable film formation. The thermal cycles of each binder are described in Table 2. From 100 °C to the temperature plateau indicated, the heating ramp used was 5.0 °C/min. The final thickness of the coatings was measured using an inductive gauge thickness measurement device Elcometer (Manchester, UK) 456, achieving values of 25 +/− 5 micron for all formulations.

### 2.3. Physico-Chemical Characterization

Thermogravimetric analysis (TGA) was performed using a Netzsch (Selb, Germany) TG 209 F1. Temperature was ramped from 40 °C to 1100 °C at 10 K∙min^−1^ under inert atmosphere (N_2_) or oxidative environment (O_2_). The gas flow rate was kept constant at 30 mL.min^−1^. The mass of samples was around 7 mg, placed in platinum crucibles. 

Differential scanning calorimetry (DSC) was performed using a Netzsch Polyma DSC214. The temperature ranged from 20 °C to 420 °C at 10 K∙min^−1^ under inert atmosphere (N_2_, flow rate 30 mL.min^−1^). The mass of samples was around 10 mg, placed in aluminum crucibles. 

Fourier transform infra-red (FTIR) analysis was performed using a Brucker (Billerica, USA) Vertex 70 FTIR equipped with an ATR module. 

X-ray diffraction (XRD) analysis was performed on a Rigaku^®^ (Tokyo, Japan) Rotaflex equipment with rotatory anode, model Rint 200, and Kα radiation of copper, with scan speed of 3 s per 0.02 θ.

### 2.4. Tribological Testing and Coating Characterization

**Figure 3:**
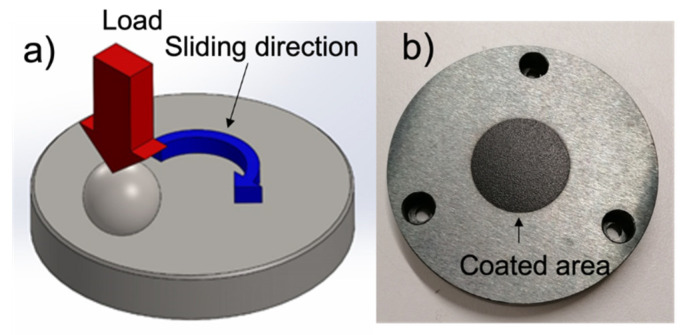
Schematic of tribological pin-on-disk contact (**a**) and a tribological specimen coated (**b**).

The tribological characterization was performed in a Bruker Tribolab system using a ball on disk configuration, following the scheme presented in Figure 3a) (high contact pressure, single point hertzian contact). The coated specimens (see Figure 3b) are the disks on this configuration. The balls used as counterface are made of 100Cr6 steel with 12.7 mm diameter from ball bearings. All the results show the average of at least two repeatable tests.

For this study two types of tests were defined:

- Tribological short-term evaluation with successive load steps of 5 N, 10 N, 20 N, and 40 N. Motion type was continuous sliding at a constant speed of 0.1 m∙s^−1^. The track radius was 10 mm and kept constant for all the samples and a sliding distance of 150 m per step, corresponding to a total of 9500 cycles at the end of the trial.
- Tribological long-term behavior were assessed using a constant pv-combination (20 N and 0.1 m∙s^−1^) and a sliding distance of 4300 m. The track radius was kept constant at 10 mm as per the incremental testing.

Friction was recorded directly by the equipment using a 2-axis load transducer, and wear was measured in terms of the position of the ball in relation to the flat disk. It is important to note that the wear measurement, in microns, includes the wear of the coating and of the steel counterface, and the thermal expansion caused by heat generated during the test. In the program, the equipment uses a load application step before the tribological test itself starts, minimizing the intrinsic elasticity effect of the rig on the wear measurement.

Adhesion testing was performed using a TQC (Nottingham, UK) cross-cut test kit CC100, following ASTM D3359 standard. The result is evaluated visually from 5B (no delamination) to 0B (extensive flaking). An internal process consisting of punching a 6 mm pin from a 0.9 mm DC04 plate coated was also used. The indenter was located on the coated side of the plate. After that, the borders of the pins and the coating itself were evaluated both visually and using optical microscopy. 

For the indentation measurements, a Zwick (Ulm, Germany) Microindenter was used. The Vickers indenter used is a Vickers V1 type made of diamond with a tip radius of 0.657 and effective opening angle of 140.6°. The Young modulus and Poisson’s ratio of the indenter are 1140 GPa and 0.07, respectively. A Poisson’s ratio for the coating material of 0.39 was assumed. All the measurements were performed considering an indentation depth not higher than 10% of the coating thickness to avoid substrate effect.

The optical microscopic images were obtained using a Digital Keyence (Osaka, Japan) Microscope VHX-2000 series, with ultra-small High-Performance Zoom Lens model VH-Z20, with magnifications from 20 to 200×.

Scanning electron microscopy (SEM) analysis was performed using a Phenom XL Scanning Electron Microscope (Thermo Fisher Scientific, Waltham, MA, USA).

## 3. Results and Discussion

### 3.1. Binders and Coatings Chemical, Physical and Mechanical Characterization

**Figure 4:**
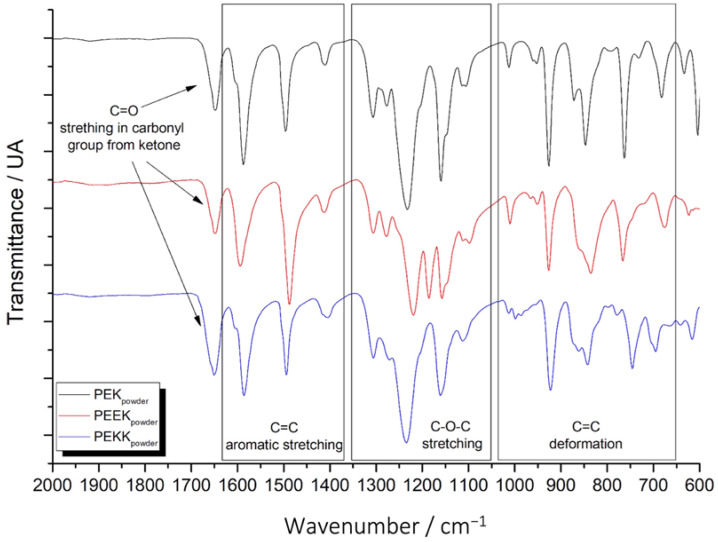
FTIR spectra of binders: PEK, PEEK and PEKK.

FTIR spectra of the materials in powder form were obtained. These are shown in Figure 4.

It is possible to identify characteristic bands that correspond to chemical groups which are common to these three polymers: C=O stretching in ketone’s carbonyl group (around 1650 cm^−1^), C=C stretching in benzene rings (region between 1400 cm^−1^ and 1600 cm^−1^), and C=C bending in benzene rings (region between 650 cm^−1^ and 1050 cm^−1^) [39,40]. The spectra show different features in these regions, due to the different arrangements of ether and ketone groups in the repetitive units of PEK and PEEK. For example, in PEK, the benzene ring always has a ketone group on one side and an ether group on the other side. In the case of PEEK, the benzene ring can be between a ketone and an ether group or between two ether groups. Lastly, in PEKK, it is possible to obtain a third case, which involves the benzene ring being surrounded by two ketone groups. This directly affects the features of the bands pertaining to the benzene rings. In the region from 1100 cm^−1^ to 1300 cm^−1^, it is possible to see a group of bands that correspond to the C-O-C ether group. In the PEEK spectrum, a band appears at around 1180 cm^−1^, which is not present in PEK or PEKK [39].

FTIR spectra (not shown here) were also obtained for the binders after being subjected to the cure treatment, showing no differences in relation to the as received powders. This indicates that curing does not seem to induce chemical modifications in the binders.

**Figure 5:**
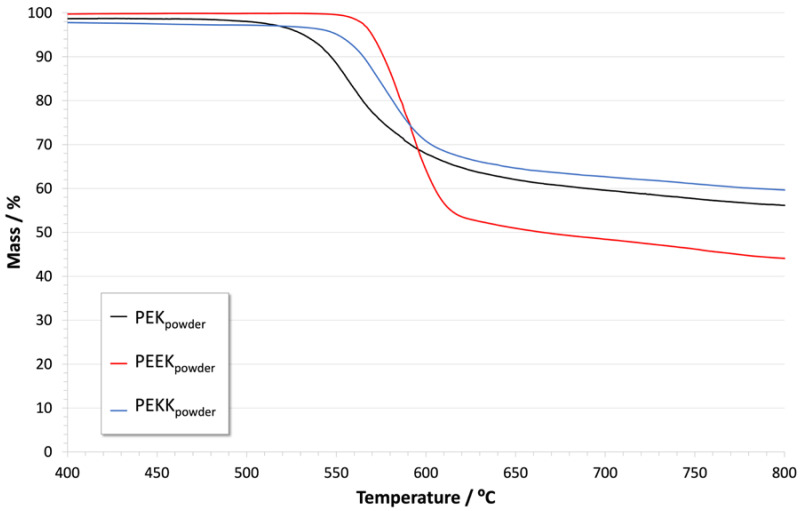
Thermogravimetric analysis of PEK, PEEK and PEKK powders in nitrogen.

**Figure 6:**
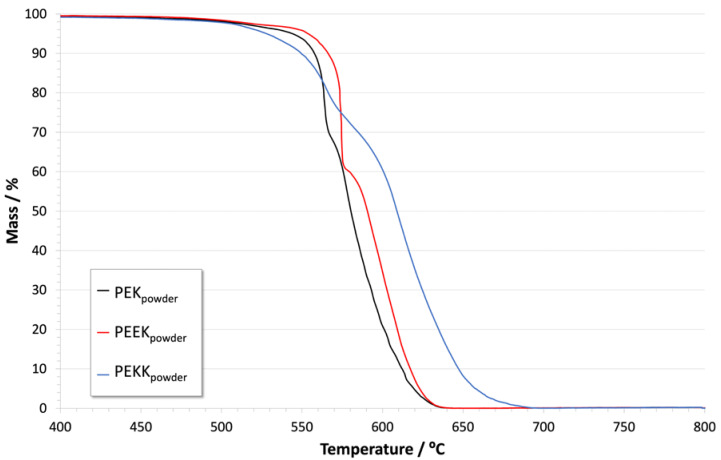
Thermogravimetric analysis of PEK, PEEK and PEKK powders in oxygen.

Figure 5 and Figure 6 show the result of thermogravimetric analysis for PEK, PEEK, and PEKK, under nitrogen and oxygen environments, respectively. Table 3 compiles the decomposition onset temperatures for each material under each condition.

As seen in Figure 5 and in Table 3, the onset of thermal degradation under nitrogen occurs between 500 °C and 600 °C for the three polymers. A major degradation step accounts for most of the mass loss, followed by slower degradation that continues up to higher temperatures. The material remaining at 800 °C corresponds to 40–60% of the initial mass. The decomposition of PEEK in inert atmosphere has been previously described as starting with random chain scission of the ether and ketone bonds, leading to production of phenols, CO, and CO_2_. This is followed by the formation of thermally stable crosslinked carbonaceous char that persists up to 1100 °C and accounts for a very significant percentage of the initial mass (about 45%) [41]. We can assume that the decompositions of PEK and PEKK follow a similar route, considering the similarity of the chemical structures. 

Under oxidative atmosphere, as seen in Figure 6, a second intense mass loss step becomes evident shortly after the first, as the char that results from initial polymer decomposition fully combusts into volatile products before the temperature reaches 700 °C. PEKK’s char shows somewhat higher stability, withstanding higher temperatures before decomposing. 

These results confirm the very high thermal stability of all the polymers, even under oxidative conditions. They are therefore not expected to be damaged by the coatings’ curing process, which takes place between 400 °C and 420 °C in air atmosphere.

**Figure 7:**
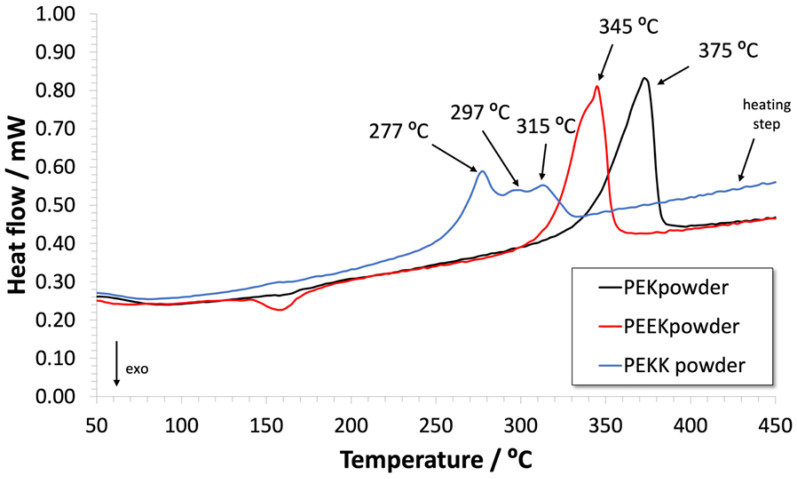
DSC thermograms for PEK, PEEK and PEKK powders for one heating cycle.

Figure 7 shows the DSC thermograms of the polymer powders for the first heating ramp. This initial heating is usually discarded, since it depends on the thermal history of the received materials, and only the thermograms corresponding to the ensuing cooling and heating ramps are considered. However, in this particular case, it is interesting to pay some attention to the features of these first heating traces. Indeed, PEKK shows a distinctly different behavior from PEK and PEEK, apparently displaying three combined melting peaks, in the temperature range between about 250 °C and 330 °C. Gardner et al. [42] reported that this complex melting behavior is a consequence of the two stable crystalline polymorphs that can occur in this polymer, one with a two-chain orthorhombic unit cell and another with a one-chain orthorhombic unit cell. Those authors observed this for PEKK prepared with a 60/40 ratio of terephthalic acid to isophthalic acid monomers, and annealed at temperatures above 260 °C for 1 h. 

**Figure 8:**
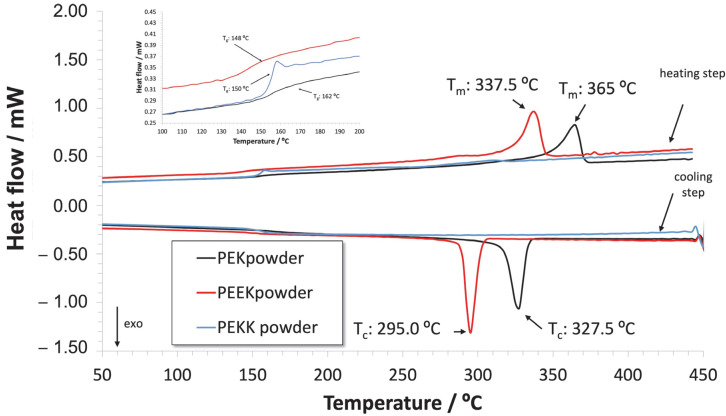
DSC thermograms for PEK, PEEK and PEKK powders. The insert shows a magnification of the thermograms for the glass transition temperature range.

Interestingly, in the subsequent cooling and second heating ramps, seen in Figure 8, PEKK does not exhibit any crystallization or melting peaks. The particular difficulty in crystalizing PEKK, which demands very slow cooling from the melt, is well known [43]. Since the industrial coating curing process involves relatively fast air-cooling rates (about 20 °C/min in controlled cooling systems), we can consider that a PEKK coating obtained industrially will possess a purely amorphous character. The other two polymers, on the other hand, display well defined crystallization and melting peaks, respectively, in the cooling and second heating ramps, thus behaving as semi-crystalline materials. Glass transition temperatures are visible in all thermograms, as seen in Figure 8, in the range of about 150–160 °C. Under the normal operation conditions of the coatings, all binders are, therefore, in the glassy state.

**Figure 9:**
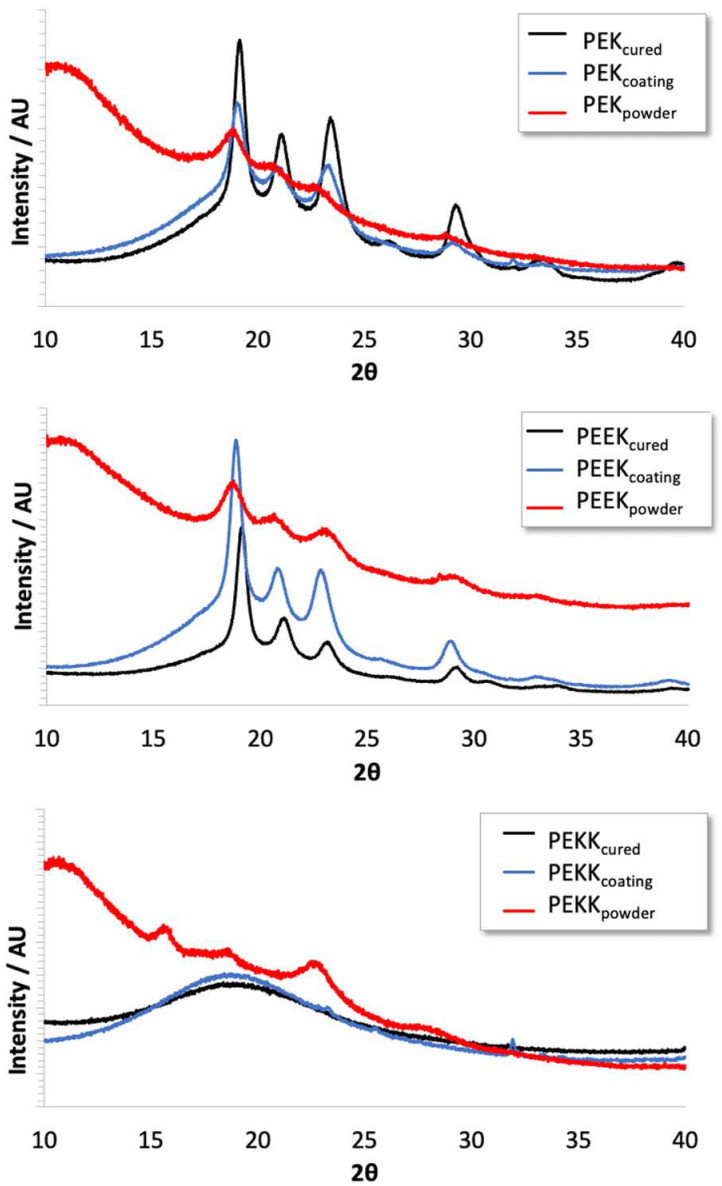
XRD spectra of polymers: powder (condition a), cured (condition b) and in coating form (condition c).

Figure 9 shows the XRD spectra for the polymers in different conditions: (a) after being subjected to a curing cycle (cured), (b) after being formulated with the coating additives, applied on a substrate, and cured to form the final coating and, lastly (c) as received (in powder form). In the coating form, PTFE powder was not added to the formulation, so as not to introduce extraneous information in the spectra.

Regarding PEK and PEEK, all materials show crystallinity bands, which are better defined after the thermal treatment. The combination of the polymers with the coating additives does not affect the resulting crystallinity. Two peaks around 18°, 22°, and 24°, correspond to the (110), (113), and (200) planes of an orthorhombic crystal structure. The 2θ peak around 29° corresponds to (213) plane of the crystal structure [42,44]. In the case of PEKK, in the “as received” powder, shows crystallinity bands. There is a 2θ peak around 16°, which is not noticed on PEK and PEEK spectra, and it is related to the (010) plane of a one-chain orthorhombic unit cell, previously reported by Gardner et al. [42] and identified as face-to-face phenyl packing. However, after being subject to the cure heating treatment, both the PEKK powder and coating formulation show an amorphous broad band, which is consistent with the results from the DSC thermograms discussed before.

**Figure 10:**
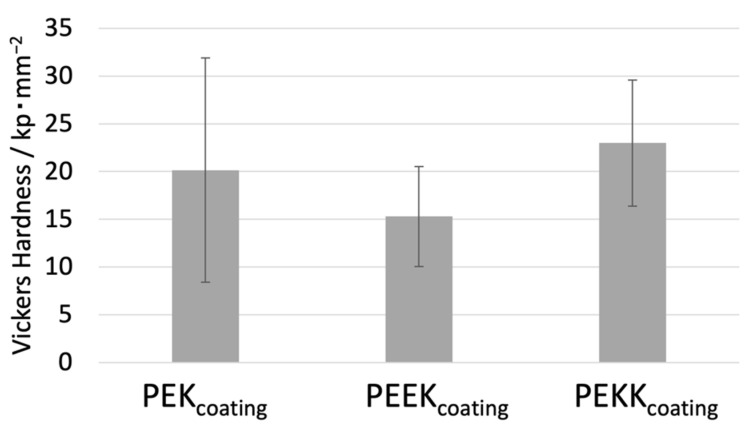
Vickers hardness measurements made in the coatings without PTFE.

Figure 10 displays the Vickers hardness values for the coating formulations without PTFE, showing that there are no significant differences between the samples. PEKK, shown to be non-crystalline, would be expected to have lower hardness. However, the glassy character of all polymers seems to be prevalent factor.

**Figure 11:**
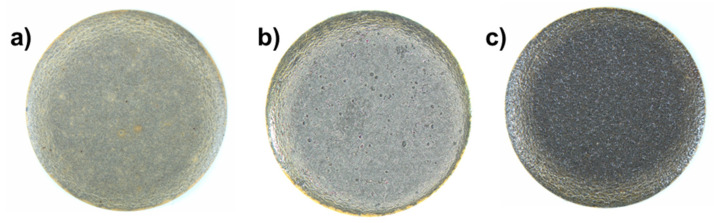
Optical microscopy images of punched 6mm pins: (**a**) PEK + PTFE_coating_, (**b**) PEEK + PTFE_coating_, and (**c**) PEKK + PTFE_coating_.

All the coatings (with and without PTFE addition) showed no detachment nor delamination in the cross-hatch adhesion test (evaluation of 5B according to ASTM D3359). Figure 11 shows the micrographs of the 6 mm pins obtained by punching the coated sheets, as described in the methods section. No delamination or flaking were noticed on the bent borders. The coatings kept their integrity in the entire area.

**Figure 12:**
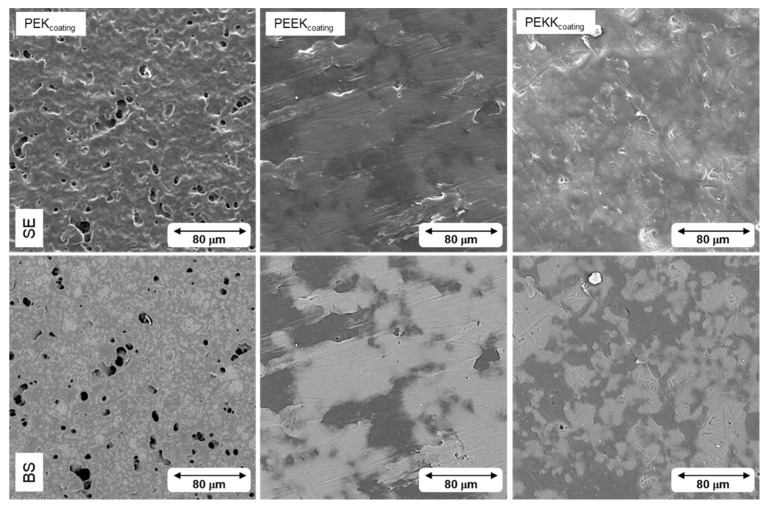
SEM images of each coating in secondary electron mode (SE) and backscattered electron mode (BS).

**Figure 13:**
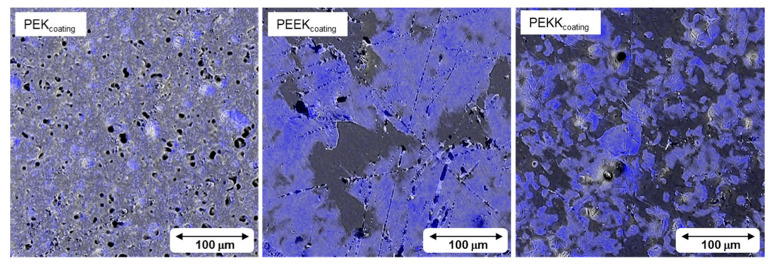
SEM images with EDS mapping of fluorine distribution (in blue) of each coating.

The coating samples used for tribological testing were inspected by scanning electron microscopy. Representative images are shown in Figure 12 (secondary electron and backscattered electron modes). EDS analysis mapping for fluorine is shown in Figure 13.

The SEM images presented in Figure 12 show that the PEK coating contains more surface defects, in the form of pinholes, than the PEEK and PEKK based coatings. These are commonly associated with air bubble retention within the coating film. Air bubbles are present in the interstices between the binder particles, after water evaporation, or are entrapped in substrate asperities. Upon curing, the binder melts, starting the particle coalescence process and formation of a continuous film. The air bubbles move towards the surface and burst, creating microcraters. These may disappear if the binder possesses sufficient fluidity to coalesce and close the pinhole [45]. The fact that PEK shows many more pinholes than PEEK or PEKK suggests that film coalescence has been hindered, probably due to higher melt viscosity, i.e., higher molecular weight of this polymer. Since no such information is available for these polymers, this hypothesis cannot be confirmed.

The distribution of fluorine on the surfaces (displayed in the EDS mappings of Figure 13) is significantly different between coatings. The lighter regions, colored blue in the EDS maps, are rich in fluoride. The PEK-based coating shows a reasonably uniform fluorine distribution, indicating that PTFE particles are well dispersed throughout the surface. PEEK and PEK, on the other hand, show regions of much higher concentration intercalated with regions of fluorine depletion. This is a symptom of incompatibility between PTFE and the binders leading to some degree of phase segregation. It must be noted that imperfect mixing always occurs in PTFE/binder systems and creates stratification during the coating coalescence process (cure), i.e., higher concentration of PTFE at the surface [46]. Fluoropolymer coatings containing binders and PTFE as self-lubricating filler are widely used industrially and the adhesion/friction non-stick properties are ensured precisely by the stratification of PTFE towards the surface (providing non-stick and low friction), whereas a binder-rich phase stays in contact with the substrate and ensures adhesion [47]. These effects seem to have been more pronounced with PEEK and PEKK, generating regions of very high PTFE concentration at the surface. However, binder-rich regions are also visible, indicating that the stratification process was not homogeneously achieved.

### 3.2. Assessment of the Tribological Behaviour

**Figure 14:**
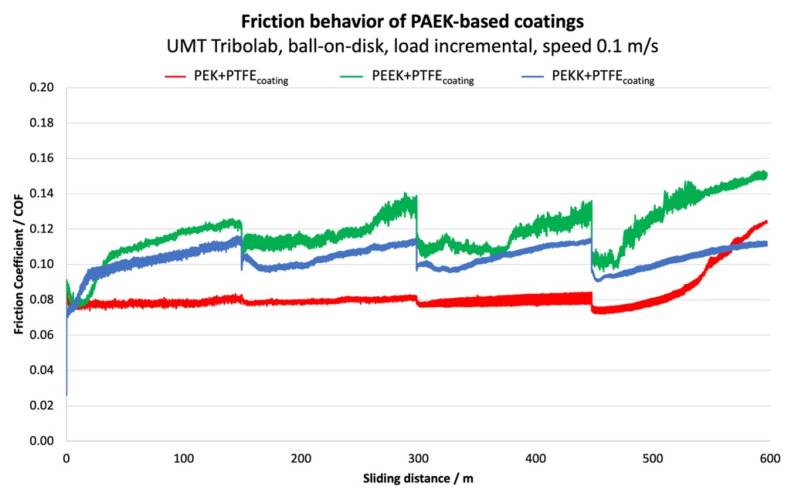
Friction coefficient of the coatings tested in incremental load conditions.

Figure 14 presents the friction behavior of the three coating formulations during the four-load incremental testing.

The PEK-based coating stands out as having very stable performance and the lowest friction coefficient. Coating failure, indicated by a significant rise in COF, is only observed during the fourth load step. PEEK and PEKK coatings show significantly different performance. COF increases steeply during an initial running-in phase. Afterwards, it increases more gradually, but never attains a stable operating value. The PEEK-based coating shows a first sign of failure during the second load step, with a sudden increase in COF at around 270 m and an increase in signal noise. In the third step, COF shows some instability, and, at around 380 m, an additional increase is observed, indicating new failure. Such a behavior can be explained by partial coating breakthroughs. However, the remaining slide-active coating islands are still capable of lubricating the contact. In step 4, COF starts increasing monotonously at an early stage, suggesting that the coating is definitely compromised. PEKK shows more stable behavior compared to PEEK. COF increases during each load step, but without sudden changes and displaying relatively low signal noise levels. The coating seems to be able to maintain its integrity and cohesion along the test, but friction is always higher than for PEK. 

The low starting friction of PEEK and PEKK coatings can be attributed to PTFE rich surfaces because of the PTFE filler stratification within the polymer matrix. Wear of the coatings causes a change of the PTFE-matrix-ratio. The initial PTFE-rich surface is worn, revealing the underlying coating with a higher portion of binder material, which has a detrimental impact on the friction performance because of more adhesive interactions. In PEK, PTFE is apparently more homogenously distributed within the binder, resulting in a very uniform friction characteristic throughout the entire test duration. The fact that PEKK is amorphous, as discussed before, can lead to different interaction between binder and PTFE particles, resulting in different tribological performance (e.g., different transfer film forming capabilities—such topics are currently under exploration).

**Figure 15:**
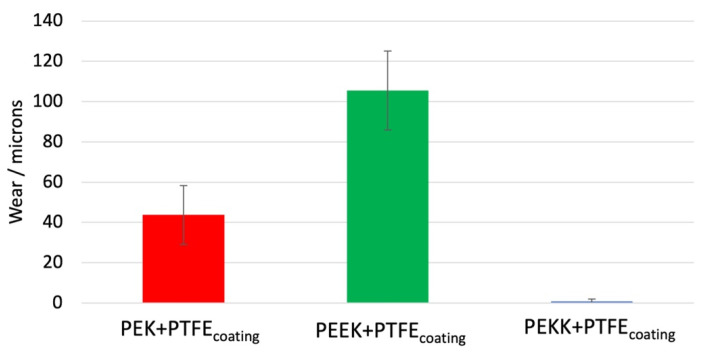
Combined final wear of the coatings tested in incremental load conditions.

Total online wear (wear = wear of coated sample + wear of the ball + thermal expansion) for each material pair is depicted in Figure 15.

Almost no wear is observed for the PEKK coating, in agreement with the relatively stable friction characteristic. The wear values obtained for PEK and PEEK are also consistent with the friction performance. The PEEK-based coating shows the highest wear, which can be associated with a complete coating breakthrough, since the initial coating thicknesses were in the range of 20–30 µm. When the coating fails, the contact transforms from a polymer-steel-contact into a steel-steel contact. A steel–steel pair is very prone to adhesive interactions building the nucleation for the extensive wear level of approximately 105 µm measured for the PEEK coatings-steel pair. Such high wear is also indicative of an early coating breakthrough (first indications of partial coating failure were noticeable in load step 2 → friction increase + instability). PEK shows intermediate wear values. The wear level is again suggestive of coating failure (initial coating thickness is 20–30 µm), which interrelates with the alterations observed in the friction signal. The lower wear compared to the PEEK coating steel pair can be explained by the later failure of the coating (PEEK coating starts to fail in load step 2, complete failure occurs at the very beginning of load step 4, whereas PEK coating failure begins at the middle of load step 4). This means that the transition into steel–steel contact happened later.

**Figure 16:**
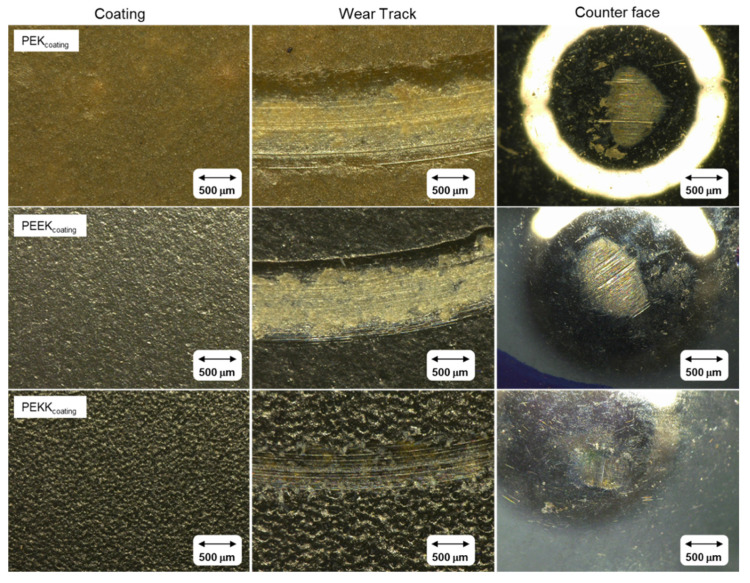
Optical microscope images of each coating and counter face tested: not-worn coating, wear track and counterface.

Figure 16 depicts optical microscopic images obtained for the coating surfaces, before and after testing, as well as the ball counter face in the tribologically stressed area.

The wear tracks of PEK and PEEK based coating show extensive wear and exposure of the steel substrate. Coating failure leads to steel–steel contact involving the substrate and the counter face, causing visible damage to the steel ball. The PEKK coating, on the other hand, kept its integrity, showing no indication of failure. The surface seems to be mainly polished, with typical running-in wear marks, and without any evidence of substrate exposure. The counter face shows minor wear marks when compared with the other two cases, and no significant scratches are noticeable. 

**Figure 17:**
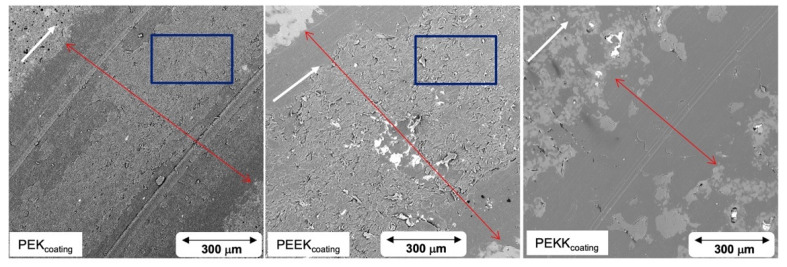
SEM images of the wear track of each coating in backscattered mode.

SEM images of the worn coating surfaces, shown in Figure 17, lead to similar conclusions. The white errors indicate the sliding direction, and the red arrows mark the wear track width.

The PEKK coating possesses the narrowest wear track width, while the widest occurs for the PEEK coating. PEKK coating is mostly intact and just shows typical running-in wear marks like a smoothing of the coating. The image also corroborates the previous statement regarding the filler gradient, i.e., the surface of the wear track appears binder-rich, explaining the higher friction. A different micro-structure is also noticeable across the entire wear tracks of the PEK and PEEK coatings, which can be linked to distinctive exposure of the metallic substrate. 

**Figure 18:**
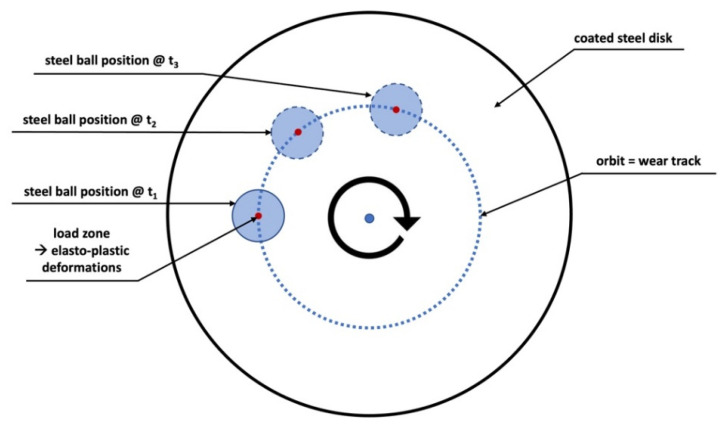
Orbit movement of the ball on the coated test specimen.

The majority of the coatings failed completely throughout testing making the assessment of the acting wear and failure mechanisms by surface inspections impossible. Nevertheless, the smooth surface of the steel ball will introduce distinctive adhesive interactions which can lead to continuous material wear but can also cause severe damage like tensile cracks due to high adhesion. The punctual load zone is moving along an orbit on the coated steel disk, as shown in Figure 18, and thus generates a reoccurring local elasto-plastic deformation of the coating which can provoke fatigue wear of the coating (cracks, crack propagation and release of loose coating fragments). Shorter tests are planned to allow for surface inspections and to gain more insights regarding the acting mechanisms that led to failure. 

**Figure 19:**
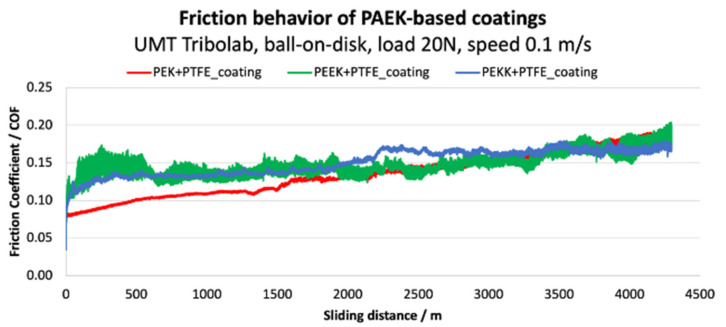
Friction coefficient average of each coating tested in a long-term testing conditions.

**Figure 20:**
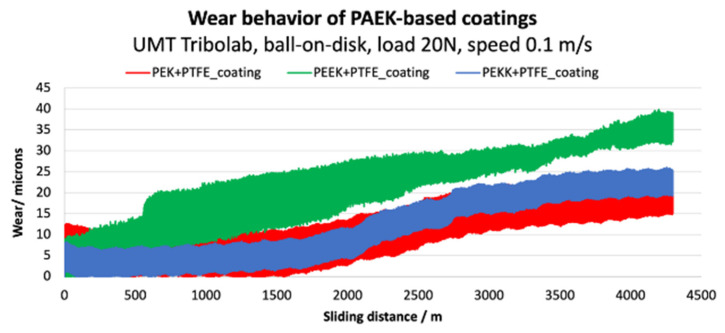
Wear behavior of each coating tested in a long-term testing conditions.

An endurance test was conducted to assess the long-term stability of the coatings under constant stress conditions (test parameters: 20 N load, continuous sliding speed of 0.1 m.s^−1^, total sliding distance of 4300 m). The results are illustrated in Figure 19 and Figure 20.

The PEK coating starts with the lowest COF and exhibits a relatively stable behavior until a sliding distance of about 1100 m. At this stage, the friction signal becomes noisier and starts to increase remarkably, suggesting coating failure. This failure is also observed in Figure 19, where at around 1500 m the wear signal starts to rise. The PEKK coating shows higher friction, which reaches a stable plateau until about 1500 m. Past this distance, COF increases until 2000 m, where more pronounced friction fluctuations become apparent. As for PEK, coating failure seems to be evidenced by the increase of the wear signal after around 1500 m. Lastly, PEEK shows significant noise and fluctuations in COF throughout the test. The wear measurement shows an increase during the first 500 m, becoming noisier and continuously increasing until the end of the test. It is important to refer that the overall wear (which comprises the coating and ball counter-face) is higher than the actual thickness (25 +/− 5 µm), confirming that the ball touches the substrate.

Overall, the endurance performance matches the incremental load test results, evidencing the better performance of PEK, in terms of lower coefficient of friction and wear. PEEK yields the most unstable behavior and higher tendency for failure and consequent substrate exposure.

## 4. Conclusions

The objective of this paper was to compare the tribological performance of coatings based on different polymeric binders from the same chemical family (PAEK). One of them, PEEK, is one of the binders most used in industrial tribological coatings. The other two, PEK and PEKK, have never been studied in coating form. 

All polymers were used in powder form and the water dispersion conditions were kept the same. It was possible to formulate stable waterborne coating formulations. The curing cycles were adapted to each binder in terms of dwelling temperature from 400 °C to 420 °C and dwelling time of 15–30 min, in order to allow formation of homogeneous and well-adhered coatings, with no chemical alterations induced by the heating process.

The main conclusions can be summarized as follows:

- PEK and PEEK show crystalline character after being subjected to the curing cycle. PEKK, on the other hand, shows some crystallinity as received from the supplier, but becomes amorphous after curing, probably due to its crystallization demanding much slower cooling conditions than the ones used in the process.
- All the binders show high thermal resistance (in both inert and oxidative environments), with the decomposition occurring above 550 °C.
- The absence of crystallinity noticed in PEKK does not impact the final coating hardness, in relation to the other binders. There are also no relevant differences in terms of substrate adhesion, with no flaking or delamination being observed in the tests.
- Distribution of PTFE particles is different throughout the different binder matrixes. There seems to be a more intense stratification effect for PEEK and PEKK, leading to higher PTFE concentration at the surface, while for PEK the particles are more uniformly distributed across the coating film depth.
- The tribological performance of the PEK-based coating evidences a persistent and stable low COF under high contact pressure, when compared with the well-known PEEK-based polymer. For PEEK and PEKK, COF increases steeply during the initial running-in phase and, in the case of PEEK, performance instability ensues, denoting coating breakthrough.
- In terms of wear, PEKK produced remarkable results, with very little wear under the test conditions when compared with PEEK-based coating produced.

The superior performances of PEK, in terms of friction, and PEKK, in terms of wear, in relation to the PEEK reference shows that these members of the PAEK family deserve being further studied in the context of tribological coatings. This will allow better optimization of coatings for specific operating conditions and/or taking advantage of lower raw material costs. One application example is the coating of automotive door hinges. PEK can be a suitable fit, allowing for smooth movement under high-pressure contact, without need for liquid lubricants. Moreover, for the automotive industry, the high-wear resistance of PEKK would be relevant for seat recliner mechanisms and seatbelt sliders.

Further studies are planned to understand the wear mechanisms involved in these binders. This will imply using test conditions that do not lead to complete wearing out of the coatings, as well as exploring other types of coating/steel contacts, e.g., pin-on-disk. 

## Figures and Tables

**Table 1:** Mechanical properties of PEK and PEEK and PEKK provided by the manufacturers.

	ASTM Standard	PEK	PEEK	PEKK
Tensile strength (MPa)	D638	90	110	110
Tensile modulus (GPa)	D638	4.3	3.5	4.0
Elongation at break (%)	D638	5	20	10–15
Flexural strength (MPa)	D790	163	173	180
Flexural modulus (GPa)	D790	4.3	4.2	4.3
Compressive strength (MPa)	D695	130	138	132
Izod impact strength (Notched) (J/m)	D256	35	53	50

**Table 2:** Curing cycle for each binder-based formulation.

	Temperature Plateau (°C)	Dwelling (Minutes)
PEK	400	30
PEEK	420	15
PEKK	400	15

**Table 3:** Decomposition onset temperatures of PEK, PEEK and PEKK in N_2_ and O_2_.

	N_2_	O_2_
PEK_powder_	535 °C	560 °C 572 °C
PEEK_powder_	570 °C	573 °C 586 °C
PEKK_powder_	558 °C	543 °C 598 °C

## Data Availability

Not applicable.
